# Barriers and facilitators to prostate cancer healthcare in Black men in the UK: from diagnosis to survivorship

**DOI:** 10.1007/s11764-025-01956-5

**Published:** 2025-12-27

**Authors:** Dena Ettehad, Hafsa Mohammed, Julia V. Bailey, Donald Schloss, Kate Walters, Samuel W. D. Merriel, William Kinnaird, Greg Shaw, Mike Kirby, Dipesh P. Gopal, Qizhi Huang, Hilary Baker, Ruth Plackett, Patricia Schartau

**Affiliations:** 1 Primary Care and Population Health Department, Institute of Epidemiology and Health Care, University College London, London, UK; 2 Black Prostate Cancer Network, London, UK; 3 Centre for Primary Care & Health Services Research, University of Manchester, Manchester, UK; 4 University College London Hospital, London, UK; 5 British Society for Sexual Medicine, Staffordshire, UK; 6 Centre for Primary Care, Wolfson Institute of Population Health Science, Queen Mary University of London, London, UK; 7 Population Health, School of Medicine and Population Health, The University of Sheffield, Sheffield, UK; 8 Centre for Primary Care, University Hospital Regensburg and University of Regensburg, Regensburg, Germany; 9 Division of Surgery & Interventional Science, University College London, London, UK; 10 Department of AI in Preventative Medicine, King’s College London, London, UK; 11 Medical Physics & Biomedical Engineering, University College London, London, UK

**Keywords:** Prostate cancer, Black men, Health inequalities, Qualitative, Barriers, Diagnosis, Treatment, Survivorship, National health service, Healthcare engagement, Healthcare experience

## Abstract

**Purpose:**

Prostate cancer (PCa) is the most common cancer among UK men, with Black men twice as likely to develop it and 2.5 times more likely to die from it than White men. This study identifies the barriers and facilitators to PCa healthcare in UK Black men and suggests ways to improve engagement and experiences across the care pathway.

**Methods:**

We conducted semi-structured interviews with:

(i) 12 UK Black men living with and beyond PCa.

(ii) 15 UK Black men aged 45 or above.

(iii) 15 UK multi-disciplinary healthcare professionals (HCPs).

Interview transcripts were coded and analysed thematically, using the socioecological model to map barriers and facilitators.

**Results:**

Barriers include mistrust of Western medicine, experiences of racism, fear and stigma around PCa, inadequate culturally sensitive information, underrepresentation of Black HCPs and Black men in healthcare spaces, limited culturally sensitive psychological support, and masculinity constructs affecting open discussions and preferences for minimally invasive treatments. Facilitators include racially concordant HCPs, culturally sensitive care, continuity of care and support from partners, peers, and local community groups.

**Conclusion:**

Our findings underscore the need for tailored culturally sensitive information, community partnerships, and Black representation in both healthcare spaces and public health campaigns to improve healthcare engagement, foster trust and improve prostate cancer outcomes for Black men.

**Implications for cancer survivors:**

Tailored healthcare for Black prostate cancer survivors is crucial. Providing culturally sensitive information, support from racially concordant HCPs and community support can significantly enhance survivorship experiences, engagement and potentially outcomes for Black men.

## Introduction

Prostate cancer (PCa) is the most common cancer among men in the UK, with a disproportionate impact on those from Black African and Caribbean backgrounds (hereafter referred to as Black men). Black men are twice as likely to develop PCa, experience more aggressive disease progression, and are more likely to die from it than their White counterparts [1–4]. They are diagnosed at a younger age (average: 67.9 years vs. 73.3 years for White men) and present to their General Practitioner (GP) at later stages [5–7]. They are more likely to be diagnosed with advanced disease, and the survival gap compared to White men continues to widen [5–7]. Despite these challenges, Black men who receive a later diagnosis are less likely to receive treatments that are proven to be their best chance of cure and report poorer healthcare experiences compared to White men [8–11].

These stark inequities prompt an examination of the underlying driving factors. While genetic factors, such as hereditary mutations in the BRCA1 and BRCA2 genes, have been identified, they do not fully explain the increased disease burden and disparities in care experienced by Black men [12–15]. Social determinants of health are key drivers of health inequities [16–18]. For PCa, socioeconomic disadvantage, knowledge gaps, risk perception, family and peer influences, cultural perceptions of masculinity, stigma, mistrust of the healthcare system, unmet support needs, patient-provider relationships and communication, and clinician bias have all been implicated as contributing factors [10, 16, 17, 19–29]. For example, the National Prostate Cancer Audit revealed that Black men felt that their side effects were not adequately explained, their opinions were overlooked, and they were insufficiently involved in care decisions—all of which directly affect their experience of care [26, 30].

There is currently no PCa screening programme in the UK and its implementation remains controversial due to the absence of a reliable test for clinically significant PCa, concerns surrounding overdiagnosis, and the limited accuracy of the digital rectal examination (DRE) [31, 32]. Patient reluctance surrounding the DRE further contributes to delayed presentation [31, 32]. Consensus amongst experts highlights the need for proactive approaches for ‘screening’ for men at higher-than-average risk, including Black men over the age of 45 [33, 34].

Primary care plays a pivotal role in PCa diagnosis and management, encompassing PSA testing, referrals via the Urgent Suspected Cancer (USC) pathway, and the management of stable or successfully treated patients. Little research has examined Black men’s experiences across this continuum or captured the perspectives of healthcare professionals (HCPs), and the nuanced influence of systemic racism on these disparities remains underexplored.

The barriers and facilitators to engaging with PCa care—and the strategies to achieve positive, equitable care experiences—are poorly understood. This study aimed to identify barriers and facilitators to prostate cancer care in the UK across the full continuum of care, from symptom recognition and presentation to primary care through to diagnosis, treatment, and survivorship. It is the first study to do so using interviews with both patients (including those at risk of developing prostate cancer and those with a prostate cancer diagnosis) and healthcare professionals working across the UK prostate cancer care pathway. The findings offer actionable insights to improve PCa care and patient experiences, with relevance to other conditions in which Black communities face disparities [35].

## Methods

Ethical approval for this study was obtained through the NHS Health Research Authority. Project ID: 323667. Reference: 23/LO/0873.

### Study design

This multi-site qualitative study explored the barriers and facilitators influencing UK Black men’s engagement with PCa healthcare, and how these could be addressed to improve engagement and care experiences.

We conducted semi-structured interviews with:

(i) UK Black men living with and beyond PCa;
(ii) UK Black men aged 45 or above without a diagnosis;
(iii) UK multi-disciplinary healthcare professionals (HCPs).

### Sampling, eligibility and recruitment

Patient participants were purposively sampled to ensure diversity in ethnicity, geographic residence, educational attainment, disease stage, and treatment modality. Primary and secondary care HCPs were purposively sampled to represent all facets of PCa care. The eligibility criteria were as follows:

- **Treatment group**: UK Black men aged 18 years or over who had received a PCa diagnosis in their lifetime and were either actively undergoing treatment or were in remission for no more than five years.
- **Screening group**: UK Black men aged 45 years or over who were eligible for PCa opportunistic ‘screening’, without a PCa diagnosis.
- **Healthcare Professional group**: National Health Service (NHS) clinical staff involved in the care of Black men at any stage of their PCa journey.

We recruited patients via Participant Identification Centres from Clinical Research Networks in North Thames, South London, and University College London Hospital, targeting areas with higher Afro-Caribbean populations. Additional recruitment leveraged social media (X), PCa charities, and PCa support groups. We recruited HCPs through professional networks, social media, and a dedicated study information page on the UCL website.

### Data collection

We conducted semi-structured interviews between June and September 2024. Interviews were held online via Microsoft Teams or in person, depending on participants' preferences. Patient interviews, lasting up to 90 minutes, explored experiences across the care pathway, perspectives on PCa and its care, and views on the factors contributing to PCa inequities. HCP interviews, lasting up to 60 minutes, focused on experiences and perspectives on care for Black PCa patients. A Black African researcher (HM) conducted patient interviews to support trust and openness, while a GP resident doctor in training (DE) conducted some HCP interviews to enable professional familiarity and clinical depth. All interviews were conducted in English, recorded and transcribed. Recruitment continued until information power was achieved [36].

### Data analysis

We conducted thematic analysis using Braun and Clarke’s six-step framework [37]. Data were coded using NVivo software (Version 14). Initial inductive coding was performed on three interviews by DE and HM, with input from JB and PS to ensure consistency and rigour. The codebook was developed iteratively and reviewed during regular team meetings. HM coded the remaining interviews. After three rounds of analysis, we mapped emerging themes onto an adapted socioecological model to contextualise findings across the individual, interpersonal, community, societal, and historical levels [38].

### Patient and Public Involvement (PPI)

We collaborated with four Black men living with and beyond PCa. PPI activities included developing the protocol, steering group meetings, co-developing information sheets, consent forms, and interview topic guides, supporting the interpretation of findings, co-authoring the paper, and supporting dissemination. Our steering committee included our PPI members, clinicians, researchers and policy makers working in the field of PCa.

### Theoretical frameworks

We used the socioecological model to examine how individual, interpersonal, community, societal, and historical factors contribute to PCa inequalities for Black men [38, 39]. We identified barriers and facilitators across these five levels, focusing on three types of racism, and their potential impacts on healthcare experiences and outcomes [40]. This study drew on the following definitions of racism, adapted from Jones et al. and Braveman et al. [40, 41].

- **Internalised racism:** The acceptance by individuals of the negative beliefs and stereotypes regarding their own racial group, i.e. self-doubt, sense of inferiority.
- **Interpersonal racism:** Occurs in interactions between individuals, where prejudice, discrimination, or negative assumptions are expressed toward a person based on their perceived race.
- **Systemic racism**: Interconnected systems, policies, practices, and norms embedded within institutions and societal structures that create and perpetuate racial inequities —encompassing structural and institutional racism.

These forms of racism intersect at every level of the socioecological model to influence PCa care and care experiences.

We define cultural sensitivity in healthcare as understanding the diverse needs of patients—shaped by intersecting identities such as ethnic background, gender, and age—and tailoring care accordingly [42]. Cultural safety goes further by encouraging healthcare systems and providers to critically examine their own cultural influences, implicit biases, and assumptions, thereby addressing power imbalances in clinical interactions [42].

## Results

### Sample characteristics

Forty-two participants were recruited and interviewed. Forty-one interviews were held online via Microsoft Teams, and one in person.

Twenty-seven Black men (mean age: 60 years; range: 41–80 years) participated in the study, including 12 men living with and beyond PCa, and 15 eligible for opportunistic PCa ‘screening’ but without a PCa diagnosis (see Table 1 and Table 2); 17 men were non-UK born, seven were UK born, and three selected “other.” Twenty-six participants were heterosexual and one was gay. The study also included 15 HCPs: eight GPs, two consultant radiographers, two consultant therapeutic radiographers, two oncologists, and one urology cancer nurse specialist.

**Table 1:** Participant  sample characteristics

Characteristic	Screening Group (*n* = 15)	Treatment Group (*n* = 12)	Total (*N* = 27)
Age, years			
41–50	3	1	4
51–60	8	5	13
61–70	3	2	5
71–80	0	4	4
80 +	1	0	1
Ethnic Background			
UK-born Black African	2	1	3
Non-UK-born Black African	8	6	14
UK-born Black Caribbean	3	1	4
Non-UK-born Black Caribbean	1	2	3
Other	1	2	3
Region			
London	9	9	18
South East	3	1	4
South West	0	1	1
East Midlands	1	0	1
West Midlands	1	0	1
Yorkshire and Humber	1	0	1
Other	0	1	1
Educational Background			
A-levels	1	1	2
NVQ Levels	2	4	6
Postgraduate Education	7	5	12
Degree	3	2	5
School Leaver	1	0	1
Undisclosed	1	0	1
Treatment History			
Active surveillance	N/A	4	4
Hormone therapy	N/A	4	4
Surgery	N/A	5	5
Chemotherapy	N/A	1	1

**Table 2:** Healthcare professionals sample characteristics

Characteristic	Total (*N* = 15)
Profession	
GP	8
Consultant Oncologist	2
Consultant Radiographer	2
Consultant Therapeutic Radiographer	2
Urology Advanced Nurse Practitioner	1
City	
London	6
Surrey	2
Exeter	1
Liverpool	1
Wolverhampton	1
Hampton	1
Nottingham	1
Sheffield	1
Manchester	1

These findings include Black men living with or beyond PCa and those without a diagnosis, who were eligible for opportunistic ‘screening’, to capture a broad range of perspectives. Participants without a diagnosis often drew on observations from loved ones or community involvement, enriching the data with their insights. Consequently, our results reflect both the personal experiences of Black men and perceptions within their communities.

### Findings

**Fig. 1:**
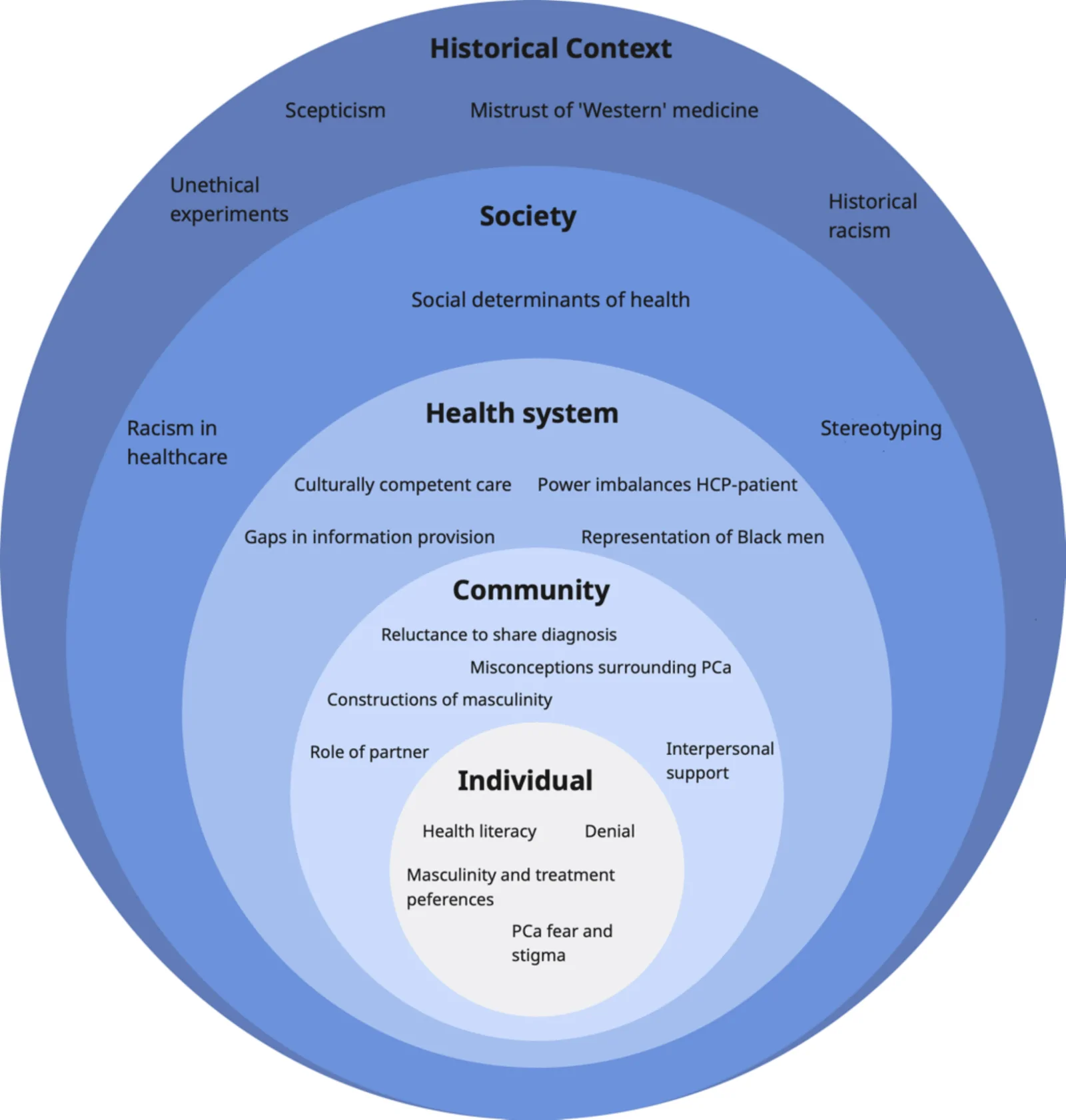
Socioecological Model: Factors contributing to inequalities in prostate cancer care for Black men

Our findings are organised according to the socioecological model which captures barriers and facilitators across five levels: historical, societal, healthcare system, interpersonal and community, and individual (Fig. 1). Internalised, interpersonal and systemic racism interact across all these levels. Overall, there was broad agreement between HCPs and patients on these themes, with any discrepancies or unique viewpoints highlighted.

### Historical context: Legacies of racism fostering mistrust

#### Mistrust of western medicine

Mistrust in Western medicine, due to historical and ongoing racism and awareness of unethical practices against Black communities, hinders Black men's engagement with healthcare.

“*And the facts are true, about research on Black people. It goes way back to South Africa and citizen stuff there. I’ve seen in America, the Tuskegee Experiment.*^1^* And have you also heard of Henrietta* *Lacks*^2^*?” - ‘Screening’ Group Participant (51–60 years old)*

Participants expressed scepticism regarding PCa statistics for Black men, believing they were exaggerated to incite fear and diminish self-esteem.

*"I believe there is mistrust in the statistics quoted about prostate cancer. I have heard that prostate cancer isn't prevalent in Africa, why are we at high risk here? There is a perception that statistics for ethnic minorities are designed to break one's self-esteem and affect confidence." - Black Afro-Caribbean HCP*

Black men reported fear of hospitals, reluctance to join clinical trials, concerns about treatment safety, and mistrust of healthcare providers’ motives. For example, one participant's father, who was diagnosed with PCa, did not want to receive treatment:

*"The thought of going to hospital is something he doesn't like. He doesn't like any form of treatment." - ‘Screening’ Group Participant (61–70 years old)*

Participants also noted the generational nature of medical mistrust:

*"I think this is a historic thing that's been passed down through generations, the mistrust of medical people in terms of research and being experimented on. Anything experimental, like clinical trials, we don't have that uptake, do we?" - Urology Advanced Nurse Practitioner*

*"The mentality of Black people, having gone through slavery where they are punished, they are oppressed, it's still at the back of the Black people's mind" - ‘Screening’ Group Participant (61–70 years old)*

One Black Afro-Caribbean HCP reflected on their personal experience with the COVID-19 vaccine, highlighting that mistrust exists even among clinicians within the Black community:

*“It took me about three months or so before I was comfortable to be vaccinated for COVID-19 because even myself as a clinician, I also have that mistrust as a Black male. I was happy to take it once I was able to do my own research, but it still took me three months compared to my Caucasian counterparts.” - Black Afro-Caribbean HCP*

Previous experiences in care fuelled mistrust:

*"I had negative experiences in hospitals...if I had an alternative, I would definitely pick the alternative rather than going into hospitals... Not every doctor is a healer—some are killers rather than healers." - ‘Screening’ Group Participant (45–50 years old)*

In some instances, this also led to a preference for alternative therapies or seeking care in countries of origin.

*“I had a guy who was over from Nigeria, and he was very concerned about healthcare here and didn’t know whether to have treatment here or go home and have treatment. You could tell at the start of the appointment that there was a lot of apprehension and fear” - Consultant Therapeutic Radiographer*

### Societal influences: Systemic racism and social determinants of health

#### Social determinants of health influencing PCa care

Social determinants of health—particularly educational attainment, interpersonal racism, health literacy and socioeconomic disadvantage—were frequently identified by participants as factors influencing PCa care access and experiences for Black men.

*"Most black men are in marginalised and deprived communities, and these communities do not receive good healthcare to begin with" - PCa Patient (51–60 years old)*

*"HCPs assume people will read medical and technical language information, but they're not. Some Black men we've talked to have low education levels" - ‘Screening’ Group Participant and Community Advocate (51–60 years old)*

Socioeconomic disadvantage shaped treatment choices. Participants described how financial instability, particularly among men in zero-hour contracts or insecure employment, influences treatment engagement and choices, with some opting for less invasive or non-curative treatments to minimise time off work.

*"I had a patient who was a builder and the main earner for his family. He wasn't going to be able to lift anything heavy for the next two to three months after his prostatectomy, and he was worried that this would affect his work—which might mean he might lose his job. As a result, he wasn't engaging with the treatment." – Consultant Oncologist*

Some Black men felt direct experiences of racism negatively influenced their care. One participant, who requested medication-based treatment, was denied this and only offered surgery.

*"It's racial discrimination, because if I were White, I would be treated nicely, but because I'm Black you didn't treat me as I'm supposed to be treated" - PCa Patient (51–60 years old)*

Although most Black men identified racism as primary contributors to PCa disparities, a few HCPs attributed differences to genetic predispositions.

*"I don't know any inequalities per se. I know Black people generally don't get diagnosed as much as they should because they are at a higher prevalence." - General Practitioner*

*“I’m sure it’s a genetic link and I also know, and I do see this quite regularly is that quite often they do present with slightly more advanced disease at diagnosis.” - Consultant Therapeutic Radiographer*

#### Reactions to diagnosis and stereotyping

Upon receiving a PCa diagnosis, many participants described feeling shock, denial, or fear. Some explained that cultural differences in emotional expression may lead healthcare professionals to misinterpret distress as aggression rather than recognising it as fear or uncertainty. This theme was primarily raised by Black or mixed-race HCPs, alongside Black patients. Language barriers, accents, and unfamiliar medical terminology further contribute to this communication gap.

*“People are afraid, and that fear can come across as anger. I’ve spoken to men about their MRI report, and they seem angry, but it’s fear—fear of the unknown, fear of what’s coming next. It’s not personal to the healthcare professional; it’s a need for support”.—Urology Advanced Nurse Practitioner.*

*“Also, when a Black man is talking, most of the time their voices are raised. It’s not like they are angry. It’s just a natural way of expression.” – ‘Screening’ Group Participant (51–60 years old).*

### Health system disparities: navigating information gaps and structural inequities

#### Gaps in information provision

Both patients and some HCPs noted significant information gaps throughout the PCa healthcare pathway, from referral to follow-up. A lack of clear, culturally sensitive information led to confusion, perceptions of inequity, and treatment regret.

At referral, some Black men were unaware of their own PSA levels, the implications of elevated results, or the urgency of a two-week referral, leaving them unprepared for their Urology appointment and subsequent diagnosis:

*"I have people turn up in my clinic with PSAs in the hundreds, and they never knew until they're sat in front of me." – Consultant Oncologist.*

Black men perceived disparities in consultation quality, noting that White patients received more thorough explanations and longer consultations:

*"The hospital called my (White) friend for a three-hour consultation on side effects before his treatment started. I didn't get any of that." – PCa Patient (51–60 years old)*

This perspective was reinforced by a Consultant Therapeutic Radiographer:

*“If English isn’t the patient’s first language or they don’t speak any English whatsoever, they’re the patients that HCPs just do not reach out to. They don’t spend enough time with them, they don’t sit and chat with those patients and it’s a real barrier. If there’s a barrier of colour, you see that the health professionals do not spend as much time” - Consultant Therapeutic Radiographer*

This information deficit contributed to treatment regret, disempowerment, and diminished confidence in the healthcare system. Black men also felt their concerns about treatment risks and side effects were dismissed.

*"As far as I was concerned, it was second-class treatment by the doctor to me. I don't think he had any empathy towards me or respect...He said to me oh we will have it out (surgery). So, I replied to him well my brother had PCa and he had radiotherapy and that’s what I want" – PCa Patient (45–50 years old)*

*"I had to really push for more answers about the side effects. I had to do my own research, and it became a mental block for me." – PCa Patient and Community Advocate (51–60 years old)*

These gaps extended into survivorship, with Black men facing unmet needs after treatment, and unclear follow-up and discharge plans. Transition to GP-led care was challenging, as GPs were often seen as unprepared for their complex needs.

*"I needed someone to help me understand my treatment plan and follow-up, but I didn't even know who to ask. I just got lost in the system." – PCa Patient (61–70 years old)*

#### Visible representation of black men in health spaces

The absence of Black HCPs in senior healthcare roles, as well as Black men in support groups, public health campaigns, and clinical trials, emerged as a key factor influencing healthcare engagement and trust. Black men preferred HCPs from similar backgrounds, as they felt shared experiences foster understanding.

*“I was fortunate. Three of the anaesthetists were from the Black community and they reassured me… It was lovely to have that.”—PCa Patient (71–80 years old)*

Black men recommended greater visible representation of Black HCPs and Black men in awareness events, healthcare settings, and public health campaigns, to build trust and engagement.

*"When a Black man is talking to you, you feel more at home, on average if you hear them talking to you, you think it's my brother talking to me." – ‘Screening’ Group Participant (45–50 years old)*

Patients in support groups tailored to Black men also highlighted the positive impact on their wellbeing, valuing shared understanding. Those who had attended unrepresentative PCa support groups felt uncomfortable, highlighting the need for culturally tailored support spaces.

*"Within the support group I think...the White voice tends to be louder and more privileged than the Black." – PCa Patient (61–70 years old)*

*“I would feel much more comfortable with a support group for Black men. It was disappointing when I went to the group and didn’t see as many if any actually Black men there” - PCa Patient (51–60 years old)*

#### Continuity of care and cultural sensitivity

Continuity of care by HCPs who understand the social, cultural, and emotional context for Black men was a key theme. Personalised care by a familiar HCP was crucial for building rapport, enabling shared decision-making, addressing fears, managing embarrassing side-effects, and meeting Black men's unique psychosocial needs.

Participants felt there were gaps in HCP cultural sensitivity. Newly qualified clinicians were seen as more aware of PCa risks and Black men's potential medical needs.

*“I always go back to the fact that healthcare professionals need full awareness. If the awareness and education aren’t there, people won’t understand the subtleties of dealing with the specific needs of somebody from the Black community.” - PCa Patient (71–80 years old)*

*“When I was first a GP, the attitude towards prostate cancer testing was very different. It was, “Oh, there’s no point, don’t bother. The tests are dangerous, you end up with unnecessary treatment and look at all these terrible consequences of treatment.” That probably spills over into knowing more about prostate cancer, like ethnic and family risk, so it could be generational. That has changed massively over the last 15 years, so any GP in their mid-forties may need updating.” - General Practitioner*

#### Mental health and support

Black men living with PCa face psychosocial challenges, shaped by cultural masculinity norms and social marginalisation. Participants noted that stigma in Afro-Caribbean communities discourages seeking mental health support, compounded by insufficient information on available services and concerns over the cultural sensitivity of mental health support. Culturally tailored mental health support was identified as crucial for improving access to psychological care for Black men.

*“We have huge issues with psychological support for prostate cancer patients. Are we able to get it with patients where language might be a barrier, or cultures where psychological support is seen as weakness? In BAME cultures, seeking support might be seen as not being strong. Patients are not able to access it, but when they do, are there clinicians who can speak the language?” - Consultant Oncologist*

### Community and individual dynamics

#### Influence of masculinity and social stigma

Culturally rooted constructs of masculinity emphasise provider roles, virility, and fertility, which shapes how Black men approach diagnosis disclosure and treatment decisions. Fear and stigma—particularly regarding PCa's impact on physical health, community-based misconceptions, and social standing—can lead to embarrassment and hesitancy in discussing one’s diagnosis.

*"Even up to now, I tell you, my wife is the only one who knows that I was treated for prostate cancer" - PCa patient (71–80 years old)*

Misconceptions surrounding transmission and stigma can impact patients:

*"I have had patients whose wives wouldn't let them sleep in the same bed, and they were ostracised." - Consultant Radiographer*

Concerns about treatment's impact on sexual performance, fertility and work capacity sometimes led to preferences for minimally invasive approaches for Black men, despite a risk of less optimal outcomes.

*“I believe that when it comes to being a provider, there is an intrinsic stoic nature to these men. There is this notion to just take the least invasive form of treatment” - General Practitioner*

#### Role of support networks: Partners and community leaders

External support emerged as a pivotal facilitator in the PCa journey. Partners were essential for processing diagnoses, supporting treatment decisions, and advocating for patients.

*“One of the men I spoke to said, ‘I was glad my partner went because she had a list of questions when I went into shock after my diagnosis. She was able to ask additional questions.’” - ‘Screening’ Group Participant and Community Advocate (51–60 years old)*

Similarly, grassroots work with community and religious leaders was seen as essential for dispelling misconceptions, fostering open discussions and promoting peer support, thereby reducing fear and enhancing understanding of PCa risk factors and symptoms.

*“It’s just going into the hubs. The Black surgeon who was on the community van project, he understood the communities as well so that made a big impact”* - *Consultant Therapeutic Radiographer*

*“I think we can probably solve half our problems if we connected all the churches and started raising awareness in church.” - PCa patient (51–60 years old)*

## Discussion

This study explored reasons for Black men’s experiences of and engagement with PCa healthcare from referral to a specialist for diagnosis to survivorship. Our analysis reveals how racism, operating at individual, interpersonal, and systemic levels affects Black men’s experiences throughout the PCa care pathway. These inequities in healthcare engagement and experiences are likely to contribute to the disparities observed in health outcomes for this group.

Our findings highlight that historical mistrust of Western medicine, reinforced by contemporary experiences of racism, shape Black men’s perceptions of and engagement with the healthcare system. At the societal level, the social determinants of health—such as socioeconomic status (SES) and education—affect power dynamics in patient-provider interactions, influencing communication, shared decision-making, and satisfaction with care. At the healthcare system level, we found that Black men consistently reported insufficient and untailored information provision, a lack of continuity of care, gaps in cultural sensitivity of HCPs, insufficient psychological support, and experiences of racial discrimination throughout their PCa journey. These factors reinforced a preference for racially concordant providers, who were perceived as more trustworthy and relatable. At the community and individual levels, we found that misconceptions about PCa fuelled stigma and fear, limiting open discussions about the diagnosis. Support from partners and community support groups were highlighted as crucial sources of practical and psychosocial support. Our data shows that cultural norms shape constructs of masculinity, reinforcing fear and reluctance to accept or disclose a PCa diagnosis. These norms also amplify concerns about treatment side effects, leading to a preference for less invasive and at times sub-optimal treatments coupled with later treatment regret.

### Individual and community influences

Notions of masculinity, which may be partly influenced by internalised racism, negatively influence health‐seeking behaviours among Black men [27, 43]. Our data indicate that culturally rooted masculinity norms—emphasising provider capabilities and virility—shape Black men’s PCa journeys. These norms may foster hesitancy with treatment engagement and lead to reluctance to disclose a diagnosis. The dual imperative to fulfil familial provider roles and maintain virility may drive Black men to prioritise treatments that preserve physical function, especially when systemic racism results in socioeconomic disadvantages that render taking time off work unfeasible [44]. Our findings highlight the complex interplay between masculinity and treatment decision-making, underscoring that effective interventions must address broader structural factors such as socioeconomic disparities and go beyond standard cultural competency training [28].

Interpersonal networks, including family, friends, and community groups, are essential sources of information and support, especially in the context of mistrust of healthcare [45, 46]. Community groups, particularly tailored support groups for Black men, provide opportunities to raise awareness, reduce stigma and offer peer support. Recent literature has suggested that partners for Black men living with PCa often feel excluded from Black men’s PCa journeys [47]. Our data highlights the importance of partners, who frequently played an active role in offering emotional support and advocacy throughout the care pathway. Female partners often play a key role in encouraging men to seek healthcare [48–50].

### Health system and wider society

At the broader health system and societal levels, our data showed that systemic racism manifests through experiences of racial discrimination, gaps in information provision, and disempowerment, impacting Black men's PCa care.

Participants in our study highlighted experiences of racial discrimination in PCa care, which may lead to poorer physical and mental health outcomes for PCa patients [51–53]. Black men are less likely to receive comprehensive information about their diagnosis, treatment options and post-treatment care, with implicit biases contributing to this disparity [54–56]. The accounts of Black men in our study reinforced this view of insufficient and untailored PCa information provision, fostering feelings of differential treatment and increased treatment regret, which may partly explain why they sometimes receive less radical treatments than White men at similar cancer stages. This indicates that treatment preferences may be shaped by both cultural masculinity constructs and driven by a lack of culturally sensitive information. Black PCa survivors in our study also described feeling lost after treatment, when navigating complex needs and during the transition back to GP-led care with minimal survivorship planning or culturally attuned support, a finding consistent with studies conducted in the USA [57, 58].

Mistrust, rooted in historical exploitation and institutional racism, exacerbates structural barriers in healthcare linked to heightened scepticism, e.g. regarding PCa statistics, and disengagement among Black communities [59–61]. Our study demonstrates that Black men’s mistrust of HCPs and the wider healthcare system is further compounded by the negative stereotypes that some HCPs hold about them. In our study, Black and mixed-race HCPs observed that Black men’s animated communication style can be misinterpreted during clinical encounters as anger or aggression instead of being interpreted as fear, vulnerability, or a need for support. Importantly, this insight was articulated exclusively by Black and mixed-race HCPs, suggesting that lived experience and cultural familiarity may confer greater sensitivity to these forms of misinterpretation. Our findings align with evidence that racial concordance between patients and HCPs enhances trust and satisfaction, with many participants emphasising a preference for Black HCPs and culturally representative support groups [62, 63]. Mistrust extends to clinical research, where Black men are underrepresented in prostate cancer clinical trials, limiting the development of evidence-based care tailored to their needs [64, 65]. Low participation both reflects and reinforces mistrust. [24, 66]. These findings underscore the importance of increasing Black HCP representation to build trust and support engagement.

Black communities in the UK are less likely to access mental health services, a disparity linked to institutional racism, a lack of tailored support, and mistrust including fears of being detained under mental health law [67]. Participants in our study reported insufficient culturally sensitive psychological support for Black men living with and beyond PCa. Those from lower SES backgrounds and with physical health conditions are more likely to experience mental health challenges [68, 69]. This gap is especially detrimental for Black PCa patients, who often face socioeconomic disadvantage, making tailored interventions essential to address psychosocial impacts of experiencing PCa.

Our study shows that the interplay of internalised, interpersonal and systemic racism undermines the delivery of personalised PCa care for Black men, potentially contributing to delayed care-seeking, reduced treatment engagement, poorer healthcare experiences, and ultimately poorer health outcomes.

### Strengths and limitations

This is the first UK qualitative study examining barriers and facilitators for engaging with PCa healthcare for Black men from the perspectives of patients, at-risk individuals, and HCPs throughout the care continuum, including both primary and secondary care. The study's diverse participant demographics and in-depth one-to-one interviews provide a comprehensive understanding of experiences, while insights from HCPs in higher and lower SES areas highlight systemic, institutional, and interpersonal factors. Participants' advocacy and community work added valuable perspectives. The socioecological model maps barriers and facilitators to appropriate healthcare from the macro to the micro level, underscoring the significance of racism—historical, systemic, interpersonal, and internalised—in compounding these barriers.

Both researchers conducting data collection were young women, which may have impacted participant comfort in disclosing personal experiences. The sample does not reflect the broader UK Afro-Caribbean community, as 50% of participants held graduate or postgraduate degrees, nearly all were heterosexual, and no transgender individuals were included. Both Black men who have sex with men and trans women face additional barriers arising from the intersection of their racial, sexual, and gender identities, and interviewing these groups would have provided valuable insights. The absence of data on participants’ religious backgrounds restricts our ability to assess the impact of religious beliefs on their views and experiences. We also did not interview Urologists or Mental Health professionals; however, a Urologist on our steering committee contributed to the interpretation of results.

### Implications for practice

Our findings underscore the urgent need for an equitable and culturally sensitive approach that addresses barriers faced by Black men living with and beyond PCa.

We found that enhancing patient–provider communication and trust is key. Adopting tailored decision aids with language personalisation and culturally relevant imagery can help ensure Black patients receive clear and accessible information about diagnosis, treatment options, and potential side effects. Prostate Cancer UK's toolkit provides an example of such an approach [70]. Another example is Prostate Cancer Research’s Infopool toolkit, which supports informed decision-making by offering culturally representative information, including video testimonies from Black men and guidance on participating in clinical trials [71]. These types of tools may help empower and foster shared decision-making with Black men which is essential for equitable care.

Many participants suggested improved cultural competency training for HCPs, however evidence for its impact is limited at present [72]. Cultural safety training which addresses power imbalances and HCP biases, including implicit biases shaped by negative stereotypes, may promote positive experiences in care [42]. Our findings show that ensuring partner, or familial, involvement in consultations may also foster a supportive environment for discussing sensitive issues.

Our study suggests that community-based interventions offer a promising avenue for building trust with Afro-Caribbean communities, facilitated through community organisations, leaders, and partners. Evidence from initiatives like the Unique Improvements programme in Leeds (UK), where Black barbers discussed PCa in trusted settings, show that leveraging culturally familiar settings can increase awareness and trust among Black communities [73, 74]. Cancer Alliances across England can also partner with local communities to deliver targeted outreach initiatives, such as South East London Cancer Alliance’s Prostate Cancer Awareness campaign [75]. By engaging people in local settings, including barbershops, faith groups, and sports clubs, campaigns can help tackle taboos and misinformation in an accessible and culturally sensitive way. Regional cancer alliances with pro-active support from GPs can serve as important bridges between healthcare systems and underserved communities, helping to embed culturally responsive care into local cancer strategies.

Our data suggests that visible representation of Black HCPs at public health events and support groups tailored to Black men can help to dispel myths and reduce stigma, promoting earlier and sustained engagement with PCa care. Improved representation in research and clinical trials can likewise improve trust in the healthcare system and treatment offered [76, 77].

Our findings indicate that addressing the psychosocial impacts of PCa for Black men is essential. Tailored mental health interventions, considering cultural norms and systemic barriers, can help Black men navigate the emotional burden of living with and beyond PCa [78–80].

## Conclusion

We found that the interplay of internalised, interpersonal, and systemic racism undermines positive patient experiences and engagement with PCa care for Black men, potentially contributing to delayed care-seeking, reduced treatment engagement, decision regret and poorer healthcare experiences, which may lead to worse health outcomes.

Our theoretical mapping highlights the need for multi-level interventions to improve access to and engagement with PCa care. By explicitly mapping barriers and facilitators from the macro to the micro level, we were able to demonstrate how the multiple forms of racism intersect with and amplify challenges throughout the care pathway. Our findings offer practical insights for service delivery improvement, policy development, and tailored community outreach. Acknowledging the role of systemic racism and tailoring care through cultural sensitivity is key for improving healthcare experiences. Implementing strategies that enhance cultural sensitivity, foster trust, and provide accessible, tailored support could help mitigate disparities in care. While the challenges are complex, interventions that address structural and interpersonal aspects of care are crucial for achieving more equitable PCa outcomes and improving healthcare experiences for Black men.

## Data sharing statement

The data that support the findings of this study are available from **University College London (UCL)**, but restrictions apply due to the use of these data under licence for the current study. As such, the data are not publicly available. However, they can be accessed from the authors upon reasonable request and with the permission of UCL.

## Abbreviations

PCa: Prostate Cancer
DRE: Digital Rectal Examination
PSA: Prostate Specific Antigen
SES: Socioeconomic Status
HCPs: Healthcare Professionals
NHS: National Health Service

## Footnotes

1 The Tuskegee Syphilis Study (1932–1972) involved unethical observation and denial of treatment to Black men with syphilis.

2 Similarly, Henrietta Lacks, a Black woman, had her cancer cells taken without consent.
